# Supplementary material for: Complexome profiling on the Chlamydomonas lpa2 mutant reveals insights into PSII biogenesis and new PSII associated proteins
Source: J Exp Bot. 2021 Aug 26;73(1):245–62. doi: 10.1093/jxb/erab390 (PMC8730698; doi:10.1093/jxb/erab390)
Supplement: erab390_suppl_Supplementary_Dataset_S1 [file erab390_suppl_supplementary_dataset_s1.zip › Supplemental Dataset 1 - Excel List and all profiles/plots/MGE1_Cre08.g370450.html]

Trivial name: MGE1  
  
Euclidean distance: 33039.18  
Mean Intensity (WT): 6706.96  
Mean Intensity (Mut): 5620.36  
Distance: 4.93  
  
MapMan: protein.co-chaperones;protein.folding.chaperones and co-chaperones.co-chaperones;protein.folding  
  
p value of intensity sums Welch test: 0.7249
